# Quinofumelin (Pesticides)

**DOI:** 10.14252/foodsafetyfscj.D-24-00009

**Published:** 2024-06-28

**Authors:** Food Safety Commission of Japan

## Abstract

Food Safety Commission of Japan (FSCJ) conducted a risk assessment of quinofumelin (CAS No. 861647-84-9), a quinoline fungicide, based on submitted documents. The data used in the assessment are fate in plants (including paddy rice and tomatoes), residues in crops, fate in livestock (goats and chickens), residues in livestock products, fate in animals (rats), subacute toxicity (rats, mice, and dogs), chronic toxicity (dogs), combined chronic toxicity/carcinogenicity (rats), carcinogenicity (mice), acute neurotoxicity (rats), subacute neurotoxicity (rats), two-generation reproductive toxicity (rats), developmental toxicity (rats and rabbits), and genotoxicity. FSCJ specified an acceptable daily intake (ADI) of 0.03 mg/kg bw per day, and consequently specified an acute reference dose (ARfD) of 0.3 mg/kg bw per day after applying a safety factor of 100 based on the NOAEL.

## Conclusion in Brief

Food Safety Commission of Japan (FSCJ) conducted a risk assessment of quinofumelin (CAS No. 861647-84-9), a quinoline fungicide, based on submitted documents.

The data used in the assessment are fate in plants (including paddy rice and tomatoes), residues in crops, fate in livestock (goats and chickens), residues in livestock products, fate in animals (rats), subacute toxicity (rats, mice, and dogs), chronic toxicity (dogs), combined chronic toxicity/carcinogenicity (rats), carcinogenicity (mice), acute neurotoxicity (rats), subacute neurotoxicity (rats), two-generation reproductive toxicity (rats), developmental toxicity (rats and rabbits), and genotoxicity.

Major adverse effects of quinofumelin were observed in the body weight (suppressed weight gain), the liver (organ weight gain and hepatocellular hypertrophy), and the large intestine (erosion/ulcer, inflammation, and hyperplasia of the mucosal epithelium in mice). None neurotoxicity, teratogenicity or genotoxicity were observed.

Although incidences of colorectal cancer were increased in male and female mice in an 18-month carcinogenicity study, the mode of action was unlikely to be genotoxic. It is therefore possible to specify a threshold dose for the assessment.

In a two-generation reproductive toxicity study in rats, findings including decreased incidence of normo-morphic epididymal spermatozoa, decreased mating rate, and extended number of days required for mating were observed in males. Prolonged estrous cycle, decreased rate of normal estrous cycle, prolonged gestation, decreased number of implantations, and decreased number of offspring were also observed in females.

Based on these results, only quinofumelin itself was identified as the substance relevant for the residue definition for dietary risk assessment in agricultural and fishery products. In livestock products, quinofumelin and total M3 metabolites (including metabolites converted to M3 by enzymatic and acid hydrolyses) were likewise identified as the substances relevant for the residue definition for dietary risk assessment.

The lowest no-observed-adverse-effect level (NOAEL) obtained from these studies was 3 mg/kg bw per day in a one-year chronic toxicity study in dogs. FSCJ specified an acceptable daily intake (ADI) of 0.03 mg/kg bw per day after applying a safety factor of 100 based on this NOAEL.

Regarding potential adverse effects of a single oral administration of quinofumelin, the lowest NOAEL value was 30 mg/kg bw per day in developmental toxicity studies in rabbits. FSCJ specified an acute reference dose (ARfD) of 0.3 mg/kg bw per day after applying a safety factor of 100 based on this NOAEL.[Table tbl_001][Table tbl_002]

**Table 1. tbl_001:** *Levels relevant to toxicological evaluation of quinofumelin*

Species	Study	Dose (mg/kg bw/per day)	NOAEL (mg/kg bw per day)	LOAEL (mg/kg bw per day)	Critical endpoints^1^^)^
Rat	90-day subacute toxicity study	0, 80, 250, 1 000, 4 000 ppm	M: 16.0 F: 19.0	M: 64.1 F: 76.0	M/F: Thyroid follicular cell hypertrophy, etc.
		M: 0, 5.19, 16.0, 64.1, 256 F: 0, 6.02, 19.0, 76.0, 261			
	90-day subacute neurotoxicity study	0, 300, 1 000, 3 000 ppm	M: 61.8 F: 73.3	M: 192 F: 211	M/F: Suppressed body weight gain, decreased food intake (No subacute neurotoxicity observed)
		M: 0, 18.5, 61.8, 192 F: 0, 21.3, 73.3, 211			
	Two-year combined chronic toxicity/ carcinogenicity study	0, 80, 250, 500/750/1 000 ppm	M: 10.4 F: 4.26	M: 34.2 F: 13.5	M: Suppressed body weight gain, decreased food intake, etc. F: Suppressed body weight gain (No carcinogenicity observed)
		M: 0, 3.28, 10.4, 34.2 F: 0, 4.26, 13.5, 46.7			
	Two-generation reproductive toxicity study	0, 80, 500, 3 000 ppm	Parents and offspring PM: 4.70 PF: 6.06 F_1_M: 5.35 F_1_F: 6.67 Fertility PM: 28.3 PF: 37.9 F_1_M: 33.5 F_1_F: 41.8	Parents and offspring PM: 28.3 PF: 37.9 F_1_M: 33.5 F_1_F: 41.8 Fertility PM: 176 PF: 205 F_1_M: 225 F_1_F: 258	Parents M: Multinucleated giant ell formation in renal proximal tubules F: Increased relative kidney weight, increased absolute and relative thyroid weights, etc. Offspring Decrease of renal and thymic relative weights, etc. Fertility M: Decreased incidence of normomorphic epididymal spermatozoa, decreased mating rate, extended mating period, etc. F: Prolonged estrous cycle, decreased rate of normal estrous cycle, prolonged gestation, decreased number of implantations and decreased number of births
		PM: 0, 4.70, 28.3, 176 PF: 0, 6.06, 37.9, 205 F_1_M: 0, 5.35, 33.5, 225 F_1_F: 0, 6.67, 41.8, 258			
	Developmental toxicity study	0, 15, 50, 150	Dams: 15 Fetuses: 50	Dams: 50 Fetuses: 150	Dams: Suppressed body weight gain Fetuses: Low body weight (No teratogenicity observed)
Mouse	90-day subacute toxicity study	M: 0, 160, 570, 2 000, 4 500 ppm F: 0, 160, 570, 2 000, 6 000 ppm	M: 21.4 F: 87.9	M: 77.1 F: 305	M/F: Colonic erosion, inflammation, and hyperplasia of the mucosal epithelium, etc.
		M: 0, 21.4, 77.1, 258, 575 F: 0, 24.9, 87.9, 305, 860			
	18-month carcinogenicity study	0, 50, 300, 1 000 ppm	M: 5.46 F: 5.14	M: 33.5 F: 30.6	M: Increased mortality, colonic inflammation, etc. F: Cataract (Increased incidence of colorectal cancer)
		M: 0, 5.46, 33.5, 110 F: 0, 5.14, 30.6, 102			
Rabbit	Developmental toxicity study	0, 10, 30, 90	Dams: 10 Fetuses: 30	Dams: 30 Fetuses: 90	Dams: Abortion Fetuses: Low body weight (No teratogenicity observed)
Dog	90-day subacute toxicity study	0, 5, 20, 70, 250/140	M: 5 F: 5	M: 20 F: 20	M/F: Increased ALP, increased absolute and relative weights of the liver, etc.
	One-year chronic toxicity study	0, 3, 12, 50	M: 3 F: 12	M: 12 F: 50	M/F: Increased GGT, increased absolute and relative weights of the liver, brown pigmentation in hepatocytes, etc.
ADI			NOAEL: 3 SF: 100 ADI: 0.03		
The critical study for setting ADI			One-year chronic toxicity study (dog)		

ADI, Acceptable daily intake; ALP, alkaline phosphatase; GGT, gamma-glutamyl transferase; LOAEL, Lowest-observed-adverse-effect level; NOAEL, No-observed-adverse-effect level; SF, Safety factor

^1)^ The adverse effect observed at LOAEL

**Table 2. tbl_002:** *Potential adverse effects of a single oral administration of quinofumelin*

Species	Study	Dose (mg/kg bw or mg/kg bw per day)	Endpoints relevant to setting NOAEL and ARfD (mg/kg bw or mg/kg bw per day)^1^^)^
Rat	Acute toxicity study	F: 550, 2 000	- F: Staggering gait
	General pharmacological study (general condition)	0, 80, 400, 2 000	M/F: 80 M: Crouching and prone positions, etc. F: Decreased abdominal muscle tone, etc.
	General pharmacological study (blood pressure, heart rate)	M: 0, 80, 400, 2 000	400 Decreased heart rate
	General pharmacological study (respiratory rates and patterns)	M: 0, 80, 400, 2 000	400 Bradypnea, slowed respiratory rate
	Acute neurotoxicity study	0, 50, 200, 800	M/F: 50 M/F: Decreased arousal, ataxia, decreased body temperature, etc.
	Developmental toxicity study	0, 15, 50, 150	Dams: 50 Dams: Decreased food intake, suppressed body weight gain
Mouse	General pharmacological study (general condition)	M: 0, 89, 250, 700, 2 000 F: 0, 250, 700, 2 000	F/M: 250 M: Reduced abdominal muscle tone and heart rate, etc. F: Reduced abdominal muscle tone
Rabbit	Developmental toxicity study	0, 10, 30, 90	Dams: 30 Dams: Decreased body weight gain and food intake
ARfD			NOAEL: 30 SF: 100 ARfD: 0.3
The critical study for setting ARfD			Developmental toxicity studies (rabbit)

ARfD, Acute reference dose; NOAEL, No-observed-adverse-effect level; SF, Safety factor

-, NOAEL could not be specified.

^1)^ The adverse effect observed at LOAEL

## References

[1] Food Safety Commission of Japan. Risk Assessment Report. Quinofumelin (Pesticides) [in Japanese]. https://www.fsc.go.jp/fsciis/attachedFile/download?retrievalId=kya20230713117&fileId=210.

